# High temperature leads to a loss of isohydry in *Pinus radiata*

**DOI:** 10.1093/jxb/erag253

**Published:** 2026-05-27

**Authors:** Kritika Sharma, Ibrahim Bourbia, Faustino Rubio Pérez, Timothy Brodribb

**Affiliations:** School of Natural Sciences, University of Tasmania, Sandy Bay, Hobart, Tasmania 7001, Australia; School of Natural Sciences, University of Tasmania, Sandy Bay, Hobart, Tasmania 7001, Australia; Departamento de Sistemas y Recursos Naturales, ETSI Montes, Forestal y del Medio Natural Universidad Politécnica de Madrid, Madrid 28040, Spain; School of Natural Sciences, University of Tasmania, Sandy Bay, Hobart, Tasmania 7001, Australia; University of Illinois Urbana-Champaign, USA

**Keywords:** Evaporative cooling, heat stress, isohydry, minimal conductance, stem water potential, stomatal conductance

## Abstract

*Pinus radiata* has been characterized as strongly isohydric, which means that it tends to close stomata to maintain leaf water potential relatively constant under changing environmental conditions. However, under high temperatures, where leaf cooling may be necessary to prevent overheating, the ability to maintain this isohydric behaviour remains unexplored. Here, we examined the impact of increasing temperature (and associated needle-to-air vapour pressure deficit, VPD_needle_) on whole plant stomatal conductance (*g*_c_), canopy transpiration (*E*_c_), minimal conductance (*g*_min_), and stem water potential (ψ_stem_) in well-watered *Pinus radiata* plants. A decline in *g*_c_ was observed as temperature increased from 22 °C to 32 °C resulting in 27% stomatal closure, followed by levelling off at further higher temperatures. However, this stomatal closure was not enough to prevent an exponential rise in *E*_c_ and pronounced declines in ψ_stem_. *g*_min_ contributed 18% of *g*_c_ at 42 °C and showed an exponential rise at temperatures higher than 42 °C. Our findings suggests that high temperatures may lead to a drop in isohydry in *Pinus radiata*. This shift may be crucial to avoid overheating and damage of plant tissues but at the same time leads to more negative ψ_stem_, thereby increasing the chances of xylem embolism. The observed shift in stomatal response reveals that even in water-conservative species like *Pinus radiata*, high temperatures may compromise stomatal regulation of water potential.

## Teaser

High temperatures result in large transpiration rates due to suppressed stomatal control, leading to large declines in stem water potential in *Pinus radiata*, a strongly isohydric species.

## Introduction

Climate change is one of the biggest challenges, imposing far-reaching effects on the ecological system. Based on World Meteorological Organization (2024) reports, the global temperature record was broken in the year 2023, with the highest rise of 1.45±0.12 °C above the long term mean since the pre-industrial era (Dunstone *et al*., 2024). In this context, it is surprising that among the many studies documenting the aftermath of drought (Sanchez-Martinez *et al*., 2023), or the combined impact of drought–heat stress (Zhao *et al*., 2013; Birami *et al*., 2020; Gong and Hao, 2023; Gauthey *et al*., 2024; Hankin *et al*., 2024), very little is known about the distinct consequences of heat waves on plant survival (Allen *et al*., 2015; Margalef-Marrase *et al*., 2020; Breshears *et al*., 2021; Hartmann *et al*., 2022; Klein *et al*., 2022). As temperature continues to rise under climate change, characterizing the effects of heat on tree function is critical for predicting future ecosystem dynamics.

Most studies of heat effects on plant function have focussed on the photosynthetic response (Teskey *et al*., 2015; Guha *et al*., 2018; Bennett *et al*., 2024) with a very few exploring the stomatal response especially in terms of water potential regulation. Water potential is highly dynamic in plants and is largely influenced by transpiration rate, and hence vapour pressure deficit (VPD). Plants close stomata at high VPD, stabilizing water loss and thus attenuating the decline in water potential that could lead to xylem cavitation and damage the plant (Choat *et al*., 2018). Changes in VPD can be caused by air moisture content and temperature. Without a change in water content of the air, rising temperatures cause VPD to increase. However, the influence of high VPD driven by elevated temperature on stomatal regulation and its consequences for water potential regulation are still not well characterized (Moore *et al*., 2021). The few existing studies have reported contradictory findings, with some showing stronger stomatal regulation under high temperature prioritizing hydraulic safety (Zhang *et al*., 2022; Gong *et al*., 2023), while others show reduced control to enable leaf cooling (Urban *et al*., 2017; Drake *et al*., 2018; Chen *et al*., 2019). This means that the plants face a trade-off between water conservation and evaporative cooling required under high temperatures (Chaves *et al*., 2016; Sadok *et al*., 2021). Plants are often classified as isohydric (tight regulation of leaf water potential via stomatal closure) or anisohydric (looser regulation, allowing leaf water potential to decline as stomata stay open) (Tardieu and Simonneau, 1998; Klein, 2014), and these modes of regulation are often assumed to remain consistent within a species across varying environmental conditions. Yet this assumption remains largely untested under increasing temperature, where the need to maintain transpirational cooling to avoid overheating may shift stomatal behaviour within the same species. A strong stomatal closure is observed as a response mechanism to increasing VPD in isohydric species like *Pinus radiata*, but there is still a potential of losing water through partially closed stomata (Brodribb *et al*., 2014) or via the cuticle (Kane *et al*., 2020). Cuticular conductance can vary with temperature and between different species (Bueno *et al*., 2019; Slot *et al*., 2021; Garen and Michaletz, 2025). Studies have reported moderate rise in cuticular permeability at low temperatures and substantial increase at temperatures higher than 35 °C (Riederer, 2006; Slot *et al*., 2021; Garen and Michaletz, 2025) making it a key factor to consider while evaluating plant response under heat waves.

Pines are evergreen conifers and therefore more likely to invest resources in strategies like evaporative cooling that maintain leaf integrity. However, given the characterization of *Pinus radiata* as strongly isohydric (Brodribb and McAdam, 2013), the sustainability of this strategy at higher temperatures remains an important question. Most of the studies reporting isohydry in *Pinus* (Rodríguez-Gamir *et al*., 2019; Sharma *et al*., 2025) have been performed under a narrow range of temperature leaving uncertainty about how isohydric plants behave under heat stress when water is abundant (Bucholz *et al*., 2020). So, the question remains: will an isohydric species exposed to high temperature exhibit a decline in the stomatal conductance to maintain isohydry, or maintain isohydry and lose water through the cuticle, or sacrifice isohydry by keeping stomata open to maintain leaf temperature? Here we examine whether *Pinus radiata*, a strongly isohydric species, maintains its isohydric behaviour under elevated temperatures, which can give us a more accurate understanding of survival of this species in extremely hot environments.

## Materials and methods

### Plant material and growing conditions

The study was carried out on 19-month-old *Pinus radiata* plants cultivated from an open pollinated seedlot (‘Seed Energy Blackwood 8’) procured from Woodlea Nursery (Springfield, Tasmania). The procured seedlings were potted in Lannen 81 trays using a potting mix comprising pine bark, controlled-release fertilizer, micronutrients and MycoApply mycorrhizal inoculation. After 2 months, seedlings were re-potted in 5 litre pots in potting mix comprising 7:4 parts of fine pine bark and coarse-washed river sand along with controlled-release fertilizer. All the plants were grown under unfiltered natural light in a controlled glasshouse cell at 25/15 °C day/night temperature and 40/80% day/night relative humidity (RH) and were watered daily to the maximum field capacity during the entire growing period.

### Experimental set-up

Five healthy plants of a similar age (19 months) and size (77.9±6.1 cm) were chosen for the experiment (Supplementary Fig. S1A–C). The plants were placed in a well-ventilated customized growth chamber (with internal dimensions of 3.4×2.4×2.3 m) 2 d prior to the experiment to give them sufficient time to acclimate to the temperature and new light conditions. During these 2 d, the growth chamber was kept at 20±1 °C temperature with 10 h of light of intensity 800 μmol m^−2^ s^−1^ at the plant canopy level using an air conditioner (5KW Daikin Inverter Split System L-Series FTXS50LVMA, Osaka, Japan) and a LED lighting fixture (Lumatek, ZEUS 1000W Pro) with coverage area of 1.5×1.5 m. This photoperiod comprising 10 h day (from 07.00 h until 17.00 h) and 14 h night was followed throughout the experiment while achieving different target temperatures. A temperature/humidity probe (HMP45AC; Vaisala Inc., Helsinki, Finland) was placed at the canopy level of plants to record relative humidity and air temperature at every 1 min interval using a datalogger (CR850, Campbell Scientific, Logan, UT, USA). A soil thermocouple was employed to measure soil temperature and soil–stem hydraulic conductivity measurements (*K*_s–s_) were corrected for viscosity. Plants were exposed to five different target temperatures (22, 27, 32, 37, and 42 °C). Each of these temperature treatments were given on consecutive days starting in the morning raising temperatures from 20 °C to required temperature treatment over a period of 2–3 h. The target temperatures were achieved using an air conditioner (5KW Daikin Inverter Split System L-Series FTXS50LVMA), a heater, and a small table fan (used to circulate air). Each target temperature was maintained for at least 1–2 h until stem water potential (ψ_stem_) and canopy transpiration (*E*_c_) achieved a steady-stable state. Relative humidity was kept almost constant in the range of 30–40% at each target temperature. The plants were watered every day to full capacity throughout the experiment making sure that soil was not limiting even at higher temperatures. Soil surface was covered with aluminium foil to prevent evaporation from soil.

For each temperature treatment, ψ_stem_ and *E*_c_ were monitored simultaneously and continuously on each *Pinus radiata* plant. ψ_stem_ was monitored using needle width variation as a proxy, measured continuously every 5 min with optical dendrometers (Cavicam Co, Hobart, Tasmania, Australia) that were attached to mature needle(s) (Bourbia *et al*., 2021; Bourbia and Brodribb, 2023; Sharma *et al*., 2025). The needle width obtained was calibrated against the Ψ_stem_ measured at every target temperature using a Scholander chamber (PMS Instrument Co., Albany, OR, USA) (Supplementary Fig. S2A). The Scholander chamber Ψ_stem_ used for calibration was measured on mature non-transpiring needles covered with damp tissue paper and aluminium foil for at least an hour before measurements to prevent transpiration and allow xylem–leaf water potential equilibrium. The relationship between needle width and ψ_stem_ was always linear across all replicates (Supplementary Fig. S2A). Every morning, a measurement was taken before turning on lights (pre-dawn water potential) followed by another measurement at target temperature. The Ψ_stem_ measurement at each temperature was taken when needle width reached steady state. Steady state pertains to a condition when both *E*_c_ and the corresponding Ψ_stem_ remain steady for at least 40 min (less than 5% change) under constant target temperature, with insignificant impact from plant capacitance.

*E*_c_ of each individual plant was monitored continuously at 5 min intervals using computer-interfaced balances (model PG5002-S, Mettler Toledo) with ±0.01 g accuracy. Each pot was watered to full capacity, and excess water was drained before placing on the balances. Aluminium foil was used to cover the soil surface to prevent evaporation from soil. *E*_c_ from each plant was normalized by the projected total leaf area (m^2^). Given that there was little needle length variation (13% coefficient of variation; averaged over all plants), the total canopy area was measured using total needle count and random sampling of five needles for average leaf area. The needles were scanned using a flatbed scanner and area was calculated using ImageJ (NIH, Bethesda, MD, USA). Note that the same five plants were assessed at each temperature treatment.

Needle temperature was measured using a portable photosynthesis system, LI-6800 (Li-Cor, Lincoln, NE, USA). During measurements, the conditions in the cuvette of the LI-6800 were very carefully aligned to match the ambient conditions of light, temperature, and VPD in the growth chamber. Given the very insignificant difference in the measured needle temperature and air temperature (0.2 °C; averaged over all the temperature treatments; Supplementary Fig. S2B), needle temperature was taken as equal to the air temperature.

The Buck equation (Buck, 1981) was then used to calculate needle-to-air vapour pressure deficit (VPD_needle_) as follows:


(1) $$\mathrm{VP}D_{\mathrm{needle}}=\left(\;1-\frac{\mathrm{RH}}{100}\right)\left(0.61121\times e^{\frac{17.502T_{\mathrm{needle}}}{240.97+T_{\mathrm{needle}}}}\right),\;\;$$

where RH is the relative humidity (in %) and T is needle temperature (in °C).

Water potential gradient (ΔΨ) was calculated using difference of soil water potential (Ψ_soil_; in MPa) and stem water potential (Ψ_stem_; in MPa), inferred from needle width variations using optical dendrometers. Ψ_soil_ was assumed to be equal to the predawn water potential as the plants were well watered and under maximum humidity.

### Whole canopy diffusive conductance

*E*_c_ and VPD_needle_ measured at each target temperature were used to calculate canopy diffusive conductance (*g*_c_) in each replicate as follows:


(2) $$g_{c}=\frac{E_{c}P_{\mathrm{atm}}}{\mathrm{VP}D_{\mathrm{needle}}}$$

where *E*_c_ (mmol m^−2^ s^−1^) refers to the normalized steady state gravimetrically measured canopy transpiration at each target temperature in individual replicate, *P*_atm_ is atmospheric pressure i.e. 101.3 kPa.

### Soil–stem hydraulic conductivity dynamics measurements

*E*_c_ measured at each steady state temperature (and VPD_needle_) and corresponding daytime ψ_stem_ in each replicate were used to calculate soil-to-stem hydraulic conductance (*K*_s–s_, mmol m^−2^ s^−1^ MPa^−1^) employing Darcy’s law:


(3) $$K_{s-s}=\;\frac{E_{c}}{\Psi_{\mathrm{soil}}-\Psi_{\mathrm{stem}}\;},\;$$

where Ψ_soil_ is the soil water potential which was assumed to be equal to the predawn water potential as the soil was kept well hydrated throughout the day.

A soil thermocouple was placed at almost 10 cm depth to measure soil temperature. Across all treatment temperatures, which ranged from 22 °C to 42 °C, soil temperature for all the replicates only varied between 21 °C and 29 °C (Supplementary Fig. S2C). Therefore, *K*_s–s_ measurements were normalized to the viscosity of water at 20 °C (Korson *et al*., 1969), shown in Supplementary Fig. S3A.

### Minimal needle diffusive conductance at different temperatures

Minimal needle diffusive conductance (*g*_min_) was measured at seven different temperatures as follows: 25, 32, 37, 40, 42, 44 and 47 °C. For the measurements, five *Pinus radiata* plants used as replicates in the main experiment were watered to maximum capacity and kept in the dark overnight to ensure full stomatal closure. The next morning, three to four fully mature hydrated needles from different fascicles were taken before turning on lights, and turgid weight was measured using a precision balance followed by scanning and measuring area using a flatbed scanner and ImageJ. These were then immediately tied in a group of three to four and cut ends were sealed using paraffin wax and labelled as per replicates. A dehydrating oven (Thermoline Scientific, NSW, Australia) was set at each target temperature 30 min before starting the treatment. Air temperature and relative humidity inside the dehydrating oven were monitored every minute using a temperature/humidity probe (HMP45AC; Vaisala Inc.) and data were recorded on a logger (CR850, Campbell Scientific). The needle groups were then hung in the dehydrating oven on a string with the help of clothes pegs. The rate of water loss was determined using difference in the weights measured at regular intervals on a precision balance. The time interval between each measurement was between 2 min (at higher temperatures) and 1 h (at lower temperatures) depending upon the target temperature. The time interval between the measurements at high temperatures was significantly reduced to avoid any damage due to excessive dehydration. VPD was calculated using temperature and relative humidity measured at each target treatment (Equation 1). Relative needle water content (RWC; %) was calculated using (Barrs and Weatherley, 1962):


(4) $$\mathrm{Relative}\;\mathrm{needle}\;\mathrm{water}\;\mathrm{content}\;(\text{\%})=\frac{\mathrm{Fresh}\;\mathrm{weight}-\mathrm{dry}\;\mathrm{weight}}{\mathrm{Turgid}\;\mathrm{weight}-\mathrm{dry}\;\mathrm{weight}\;}\times 100,$$

*g*_min_ (mmol m^−2^ s^−1^) was then calculated using slope of the linear part of the needle mass–time relationship, measured needle area (m^2^) and VPD (mol mol^−1^) in each replicate (Sack and Scoffoni, 2010; Slot *et al*., 2021; Wang *et al*., 2024). This linear part was selected in relative needle water content range of 80–60% where stomata were closed and water loss occurred primarily through the cuticle, allowing accurate calculation of *g*_m_ᵢ_n_.

*g*_min_ was calculated as:


(5) $$g_{\min}=-\frac{\frac{dw}{dt}\times 1000}{\;A_{\mathrm{needle}}\times 18\times \mathrm{VPD}},$$

Here, d*w*/d*t* is the linear slope of needle mass change over time (g s^−1^) within the selected RWC interval, *A*_needle_ is the total needle area (m^2^), VPD is the vapour mole fraction deficit (mol mol^−1^), 18 is the gram molar mass of water (g mol^−1^) and 1000 converts mol to mmol.

### Measurement of maximum quantum yield of photosystem II

Maximum quantum yield of photosystem II (*F*_v_/*F*_m_) was used as an indicator of damage to photosystem II under increasing temperature from 25 °C to 52 °C. The *F*_v_/*F*_m_ ratio was evaluated by measuring chlorophyll fluorescence using a portable leaf clip MINI-PAM-II Photosynthesis Yield Analyzer (Heinz Walz GmbH, Effeltrich Germany) (Supplementary Fig. S3B). A fascicle with three to four needles sampled from each of the five replicates was kept in dark in the dehydrating oven (Thermoline Scientific) under each temperature treatment for approximately 1 h and an average from three measurements taken on same needles was used for calculations.

### Measurement of morphological traits

Morphological traits like plant height (cm), total leaf area (cm^2^) and leaf mass per area (LMA; mg cm^−2^) were measured from all the replicates after completing the experiment. Five randomly sampled needles were scanned and area was measured using a flatbed scanner and ImageJ and oven-dried (Thermoline Scientific) at 70 °C to procure dry weight. LMA was calculated as a ratio of dry weight of five sampled needles to the total surface area of needles. Total needle count and average needle area from each plant were used to calculate the total leaf area (cm^2^).

### Statistical analysis

The study analysis was done using RStudio 2024.12.0+467. Differences in ψ_stem,_  *E*_c_, *g*_c_, and *K*_s–s_ at different target temperature treatments were assessed using analysis of variance (ANOVA). A post-hoc Tukey multiple comparison of means test was employed in cases of significant differences (when *P*<0.05). Results are presented as mean values ±SD.

Various models like linear, quadratic, exponential, and cubic were deployed to study the relationship of average *g*_c_ and *g*_min_ (calculated over for all replicates) with temperature over the entire temperature treatment range (22 °C to 42 °C). A cubic polynomial model was found to be the best fit (the highest *R*^2^ and lowest Akaike information criterion values) for evaluating the relationship between *g*_c_ and temperature while an exponential model best described the relationship between *g*_min_ and temperature.

We modelled stomatal responses to determine the degree of stomatal closure required to maintain constant ψ_stem_ under rising VPD driven by increasing temperature, thereby maintaining near-isohydric behaviour, and compared this to the observed stomatal closure. We quantified how much *g*_c_ would need to be reduced to maintain a constant ψ_stem_ (hence a constant soil-to-stem water potential gradient, Δψ) set to the value observed at low temperature (22 °C) in each replicate using the following equation:


(6) $$\mathrm{Modelled}\;g_{c}=\frac{P_{\mathrm{atm}}\times \Delta \psi_{22\circ C}\times K_{s-s}}{\mathrm{VP}D_{\mathrm{needle}}},$$

Here we used the average *K*_s–s_ (normalized to the viscosity of water at 20 °C) observed across all the temperature treatments, and Δψ_22°C_ is the difference between Ψ_soil_ (soil water potential, which was assumed to be equal to the predawn water potential as the soil was kept well hydrated throughout the day) and Ψ_stem_ at 22 °C.

## Results

### Temperature-induced decline in stem water potential

**Fig. 1.**
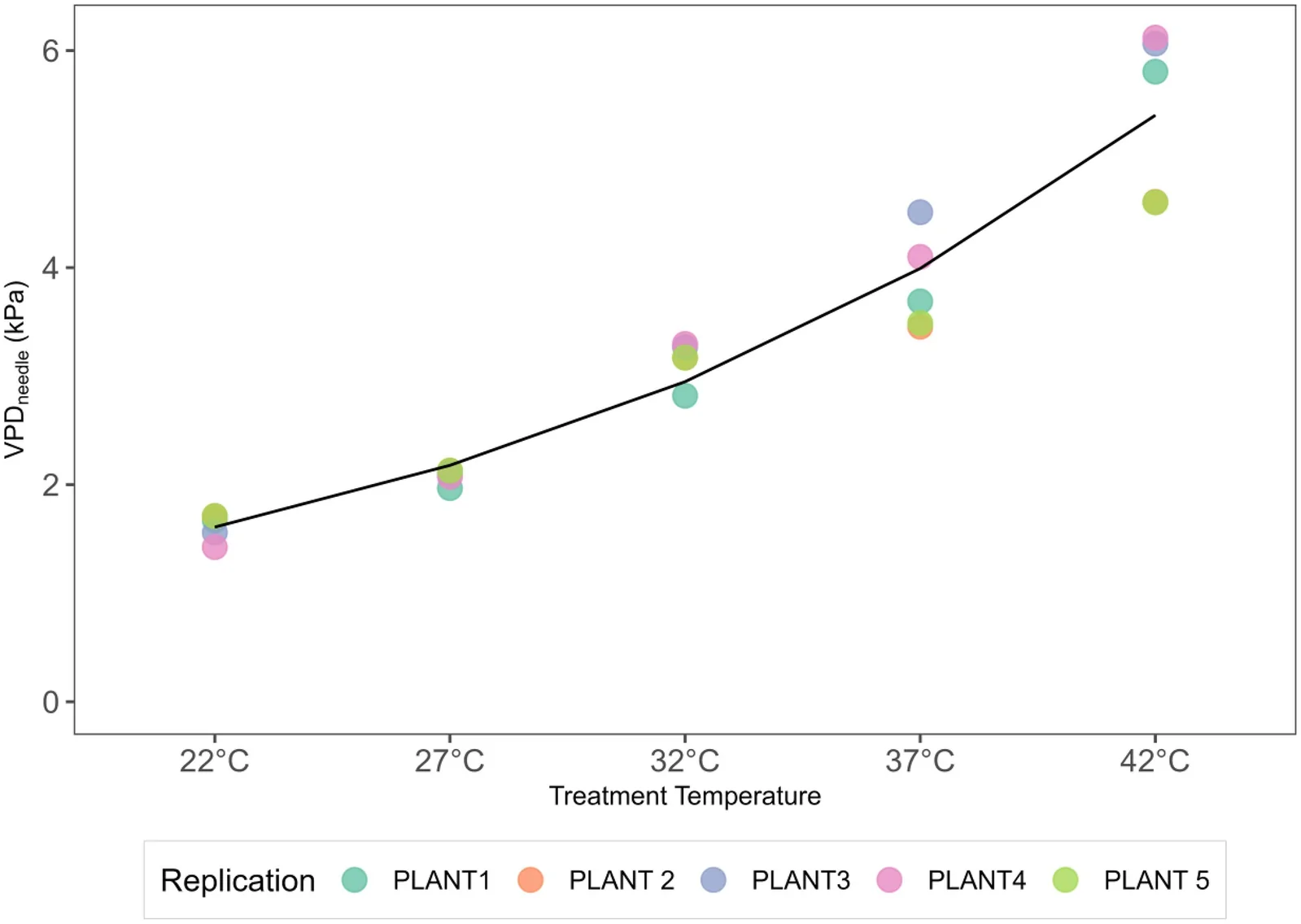
Series of temperature (°C) and corresponding needle-to-air vapour pressure deficit (VPD_needle_) (kPa) employed as five different treatments in the study. Different colours represent the replicates used.

**Fig. 2.**
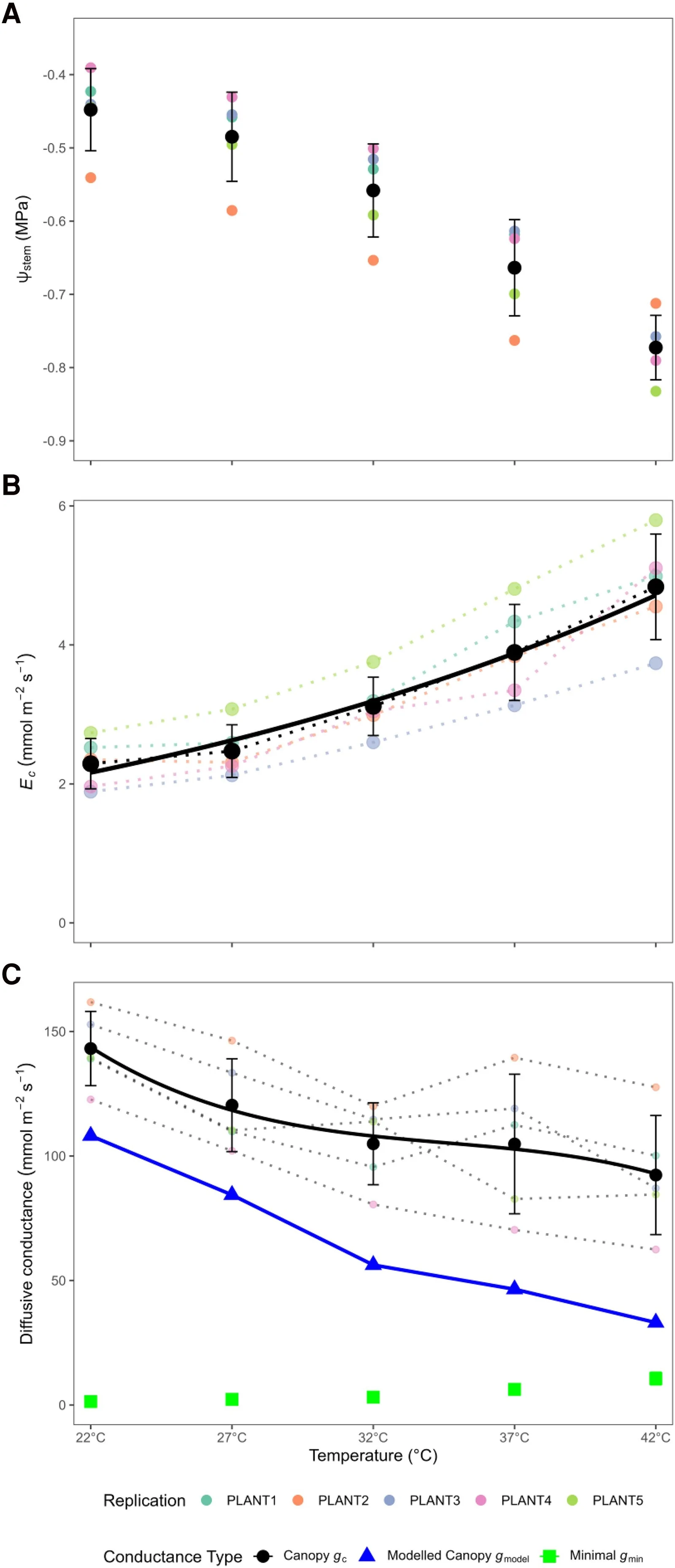
Response of mean stem water potential (ψ_stem_, MPa) (A), mean canopy transpiration (*E*_c_) (B), and mean canopy diffusive conductance (*g*_c_, mmol m^−2^s^−1^), modelled canopy diffusive conductance (*g*_model_, mmol m^−2^s^−1^), and minimal conductance (*g*_min_, mmol m^−2^s^−1^) (C) to different temperature treatments in all *Pinus radiata* replicates (represented by different colours). The black circle represents the mean of ψ_stem_, *E*_c_, and *g*_c_ and error bars show the standard deviation among all the replicates at each temperature treatment. In (B), the black solid line represents the exponential fit of response of *E*_c_ to different temperature treatments. In (C), the solid black line represents the cubic polynomial fit of the response of *g*_c_ to all temperature treatments and the blue solid line represents *g*_model_.

Each *Pinus radiata* plant was subjected to five temperature treatments from 22 °C to 42 °C and corresponding VPD_needle_ ranging from 1.4 kPa to 6.1 kPa (Fig. 1). During the temperature treatments, the mean ψ_stem_ from all the replicates declined from −0.4±0.1 MPa at 22 °C to −0.8±0.0 MPa at 42 °C (Fig. 2A). We observed a consistent decline in mean ψ_stem_ over the entire range of temperature treatments.

### Response of transpiration and canopy diffusive conductance to different temperature treatments

We observed an exponential rise in transpiration (*E*_c_) with increase in temperature (Fig. 2B). The mean *E*_c_ ranged from 2.3±0.4 mmol m^−2^ s^−1^ at 22 °C to 4.8±0.8 mmol m^−2^ s^−1^ at 42 °C.

**Fig. 3.**
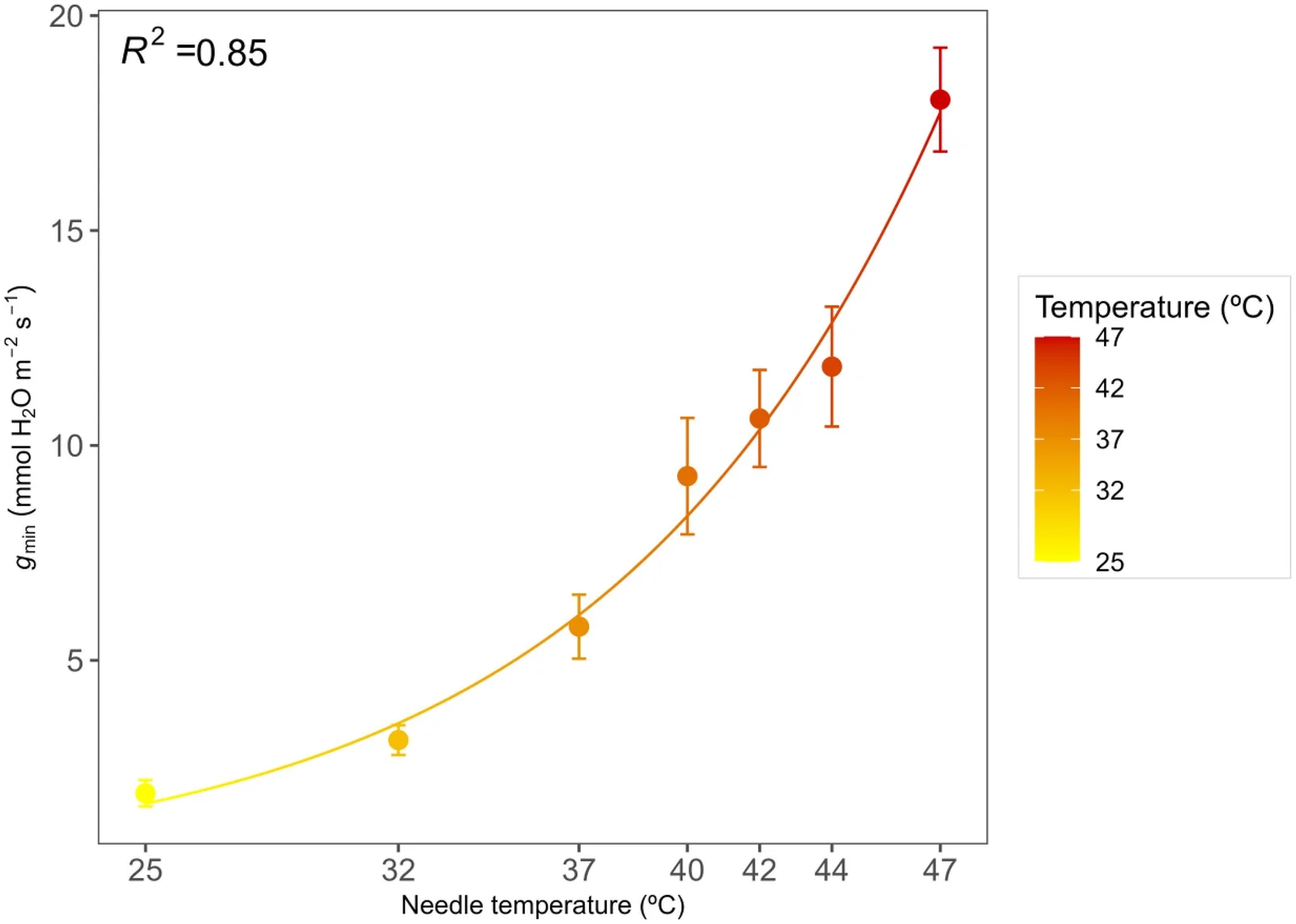
Increasing minimal conductance (*g*_min_, mmol m^−2^s^−1^) with rising temperature treatments in *Pinus radiata* replicates. The error bars represent standard deviation over all the replicates at each temperature treatment. The solid line represents the exponential fit over the response of *g*_min_ to all temperature treatments.

Stomatal response at all the temperature treatments was found to be the same among all the well-watered replicates (ANOVA, *P* > 0.05) (Fig. 2C). We observed that the overall canopy diffusive conductance (*g*_c_) declined by 27% between 22 °C and 32 °C followed by levelling off at higher temperature treatments. However, this was insufficient to prevent the continuous decline in ψ_stem_ with increasing temperatures. Based on the *g*_model_ (calculated using Equation 6), 70% reduction in *g*_c_ is required to maintain a constant ψ_stem_ as the temperature increases between 22 °C and 42 °C (Fig. 2C). An exponential rise in *g*_min_ was observed with increasing temperature (*R*^2^=0.85), increasing from 1% at 22 °C to 18.2% of *g*_c_ at 42 °C (Figs 2C, 3).

## Discussion

*Pinus radiata* is one of the most widely planted softwood conifer species in the southern hemisphere, valued for its fast growth and high-quality wood production. Due to increasing numbers of hot and dry days, large scale mortality has been observed in *Pinus radiata* growing areas of Australia, New Zealand, Africa, Chile, and USA (Carnegie *et al*., 2022). Therefore, there is an increasing need to evaluate the stomatal response under high temperatures. *Pinus radiata* has been characterized as strongly isohydric (Martínez-Vilalta *et al*., 2004), which means it maintains its daytime minimum leaf water potential relatively constant under increasing VPD or drying soil conditions due to stomatal closure (Brodribb and McAdam, 2013; Rodríguez-Gamir *et al*., 2019; Sharma *et al*., 2025). But the question that remains unanswered is whether this isohydric behaviour is maintained under conditions of high temperature where demands to maintain leaf cooling might require non-isohydric behaviour. To answer this question, we examined the impact of increasing temperature (and associated VPD_needle_) on stem water potential and stomatal conductance of *Pinus radiata* saplings. We found that stomatal conductance was not sufficiently regulated to maintain isohydry under increasing temperature, resulting in constantly rising transpiration and declining ψ_stem_.

The observed stomatal closure between 22 °C and 32 °C is consistent with the high sensitivity observed in *Pinus radiata* in previous studies (Weston and Bauerle, 2007; Brodribb and McAdam, 2013; Lahr *et al*., 2015; Sharma *et al*., 2025) and is also consistent with our study results where stomata closed by 27% as temperature increased from 22 °C to 32 °C (Fig. 2C). This stomatal control is typically observed under increasing temperature and VPD to maintain homeostasis (Weston and Bauerle, 2007; Lahr *et al*., 2015; Sharma *et al*., 2025). However, further increase in temperature to 42 °C did not lead to any additional decline in *g*_c_, resulting in a further decline in ψ_stem_. Our results are consistent with the study reporting a shift in isohydric behaviour in young apple trees in response to increasing drought stress (Zhao *et al*., 2023). Stomatal behaviour has often been interpreted through a simplified isohydric-anisohydric framework (Tardieu and Simonneau, 1998), which is often assumed to be constant within a species across conditions. However, our data suggests that isohydric and anisohydric behaviour may not be fixed, enabling species to shift water use regulation under specific conditions, such as high temperature (Guo *et al*., 2020).

A plausible explanation for the breakdown of isohydric regulation under high temperature may reflect a difference between long-term drought-induced stomatal closure and short-term stomatal responses to acute heat stress. Previous studies showing extreme isohydry in *Pinus radiata* have been performed under slow and long-term exposure to drought, where plants had enough time for abscisic acid (ABA) synthesis to reach levels sufficient to induce strong stomatal closure (Brodribb and McAdam, 2013; Rodríguez-Gamir *et al*., 2019). By contrast, in our experiment, plants were exposed to high temperature for short time period, which may not have allowed sufficient time for ABA to accumulate to levels capable of inducing near-complete stomatal closure (Mitchell *et al*., 2017; Rizzuto *et al*., 2024). These short-term diurnal temperature variations are very common under field settings where the temperature can ramp up very quickly on hot days during a heatwave. Under these conditions, stomatal behaviour may be governed more by passive hydraulic responses to declining water potential than by strong hormonal control.

Some studies have reported that stomata remain partially open at high temperatures (Weston and Bauerle, 2007), which helps to mitigate overheating of the leaves via evaporative cooling especially under well-hydrated conditions (Aparecido *et al*., 2020; Gommers, 2020). But this comes at the cost of declining water potential in the plant. The decline in ψ_stem_ shows the inability of *Pinus radiata* to hold its water potential from dropping at high temperatures, which could eventually lead to xylem embolism (Tyree and Sperry, 1989). The ψ_stem_ in our study declined from −0.4±0.1 MPa at 22 °C to −0.8±0.1 MPa at 42 °C (Fig. 2A); however, the xylem embolism observed in *Pinus radiata* roots and stem parts does not start until water potential reaches around −2 MPa (Harrison Day *et al*., 2024). This implies that there is still some space for *Pinus radiata* to keep its stomata open at the expense of declining water potential before it reaches the point of xylem embolism.

After stomatal closure, plants may use water loss via cuticle and incompletely closed or leaky stomata, together accounting for increased minimal conductance (*g*_min_) (Machado *et al*., 2021; Slot *et al*., 2021; Garen and Michaletz, 2025). Minimal conductance can vary with temperature and between different species (Duursma *et al*., 2019). Our results are consistent with studies reporting a steep rise in minimal conductance at high temperatures (Bueno *et al*., 2019; Billon *et al*., 2020; Wang *et al*., 2024), which makes it a key factor to consider when evaluating plant response under heat waves (Fig. 3). The rising evaporation rates and water loss via cuticle and leaky stomata might have prevented tissue damage at high temperature by buffering the high needle temperatures (Rehschuh and Ruehr, 2022).

Maintaining high transpiration and non-cavitating ψ_stem_ at high temperatures requires a high and constant hydraulic supply. In this species, *K*_s–s_ remained stable despite increasing air temperature (Supplementary Fig. S3A). This is in agreement with previous studies reporting constant soil–stem hydraulic conductance under well-watered conditions in different species (Hailey *et al*., 1973; Dubé *et al*., 1975; Bourbia *et al*., 2022). A small decline was observed in *K*_s–s_ value (without accounting for viscosity effect) at 42 °C but was not sufficient to induce further stomatal closure or excessive decline in ψ_stem_, as *K*_s–s_ remained high (6.8±1.3 mmol m^−2^ s^−1^ MPa^−1^; Supplementary Fig. S3A). The constancy of *K*_s–s_ as *g*_c_ declines by 36% with increasing temperature also confirms that in this species, stomatal closure is not driven by any decline in *K*_s–s_ when soil is wet (Bourbia and Brodribb, 2024).

## Conclusion

Our study suggests that high temperatures are capable of shifting the isohydric behaviour in *Pinus radiata*. This shift may be associated with lack of rapid ABA biosynthesis under short-term temperature stress, thereby avoiding overheating of plant tissue but at the cost of declining stem water potentials to levels that could increase the risk of embolism if not properly regulated. The observed shift in isohydric response provides an opportunity to understand that even species like *Pinus radiata*, which are characterized as strongly isohydric, might lose their stomatal control at higher negative water potentials. This also implies that high temperatures may accelerate mortality in all other less isohydric species, especially under soil drought conditions. Therefore, there is a need to re-evaluate the characterized response at temperatures corresponding to heatwaves to predict the repercussions of future heatwaves on the survival and growth in different plant species.

## Supplementary Material

erag253_Supplementary_Data

## Data Availability

The primary data supporting this study were not made publicly available at the time of publication. The data that support the findings of this study are available from the corresponding author upon request.

## Supplementary data

The following supplementary data are available at *JXB* online.

Fig. S1. Morphological measurements of *Pinus radiata* plants.

Fig. S2. Variations in stem water potential (ψ_stem_), average needle temperature and soil temperature across temperature treatments.

Fig. S3. Response of mean soil-to-stem hydraulic conductance (*K*_s–s_, mmol m^−2^ s^−1^ MPa^−1^), mean *K*_s–s_ normalized to 20°C and maximum quantum yield of photosystem II (*F*_v_/*F*_m_ ratio) across temperature treatments.
