# Supplementary material for: High temperature leads to a loss of isohydry in Pinus radiata
Source: J Exp Bot. 2026 May 27;77(17):5566–74. doi: 10.1093/jxb/erag253 (PMC13577784; doi:10.1093/jxb/erag253)
Supplement: Supplementary file 1 [file erag253_supplementary_data.pdf]

Supplementary figures:

Measurement of morphological and growth traits:

Supplementary Fig. S1A-C: **Five replicates of *Pinus radiata* with mean plant height of 77.94±6.11 cm, leaf mass per area (LMA) of 15.75±1.45 mg cm^{-2} and total leaf area of 1489.68±240.51 cm^{2} were chosen for the study to avoid any effects of variable growth on the stomatal response.** (A) **Representation of plant height (cm) of five *Pinus radiata* plants.** (B) **Representation of leaf mass per area (LMA; mg cm^{-2}) of five *Pinus radiata* plants.** (C) **Representation of total leaf area (cm^{2}) of five *Pinus radiata* plants.**

ALT TEXT:

Supplementary Fig. S1A-C: (A) Bar plot of plant height (cm) for 5 replicates (each shown with a different colour), demonstrating no major growth variability across replicates. (B) Bar plot of leaf mass per area (LMA; mg cm^{-2}) for 5 replicates (each shown with a different colour), demonstrating no major growth variability across replicates. (C) Bar plot of the total leaf area (cm^{2}) for 5 replicates (each shown with a different colour), demonstrating no major growth variability across replicates.

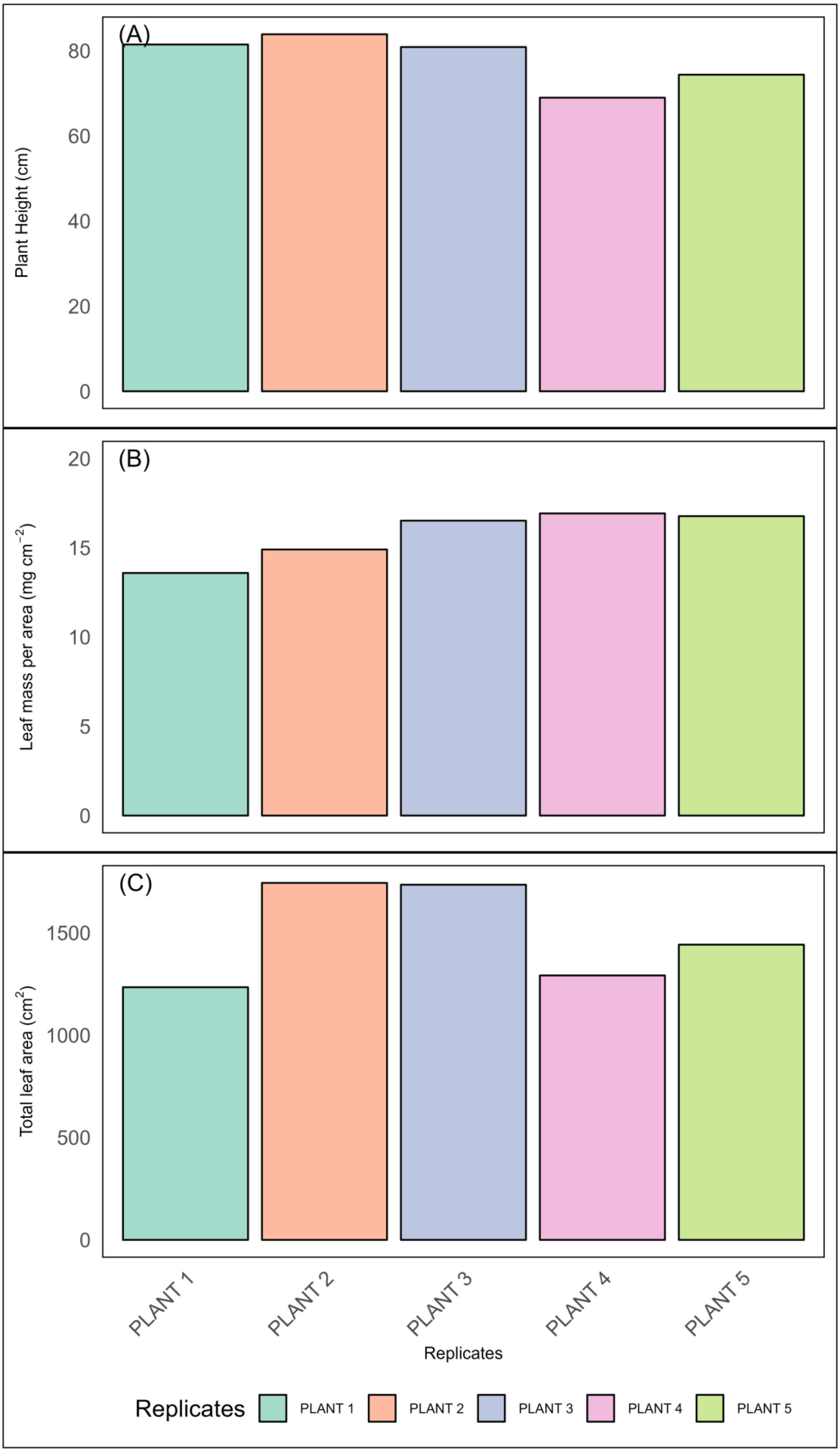

(A)
Plant Height (cm)
80
60
40
20
0
(B)
Leaf mass per area (mg cm^{-2})
20
15
10
5
0
(C)
Total leaf area (cm^2)
1500
1000
500
0
PLANT 1
PLANT 2
PLANT 3
PLANT 4
PLANT 5
Replicates
Replicates
PLANT 1
PLANT 2
PLANT 3
PLANT 4
PLANT 5

Supplementary Fig. S2 (A) **Relationship between stem water potential (Ψ_{stem}, MPa) measured using pressure chamber and normalised needle width monitored by optical dendrometers (in pixels) in *Pinus radiata* plants exposed to five different temperature treatments. Different colours in the graph represents replicates exposed to all the temperature treatments**. **Normalised needle width was found to be linearly correlated with Ψ_{stem} measured from non-transpiring needles in each replicate (with average r^2 > 0.97).** (B) **The difference between average needle temperature and treatment temperature over all the treatment groups (ranging from 22ºC - 42ºC) was found to be 0.2ºC.** (C) **Changes in soil temperature in response to increasing temperature at each temperature treatment.**

ALT TEXT:

Supplementary Fig. S2 (A) Plot showing linear relationship between stem water potential (Ψ_{stem}, MPa) and normalised needle width in five replicates (represented by different colours) exposed to five different temperature treatment. (B) Plot showing very insignificant difference of 0.2ºC between the average needle temperature and treatment temperature over all treatment groups. (C) Plot shows that the soil temperature only varied between 21- 29ºC across all the temperature treatment ranging from 22 to 42ºC.

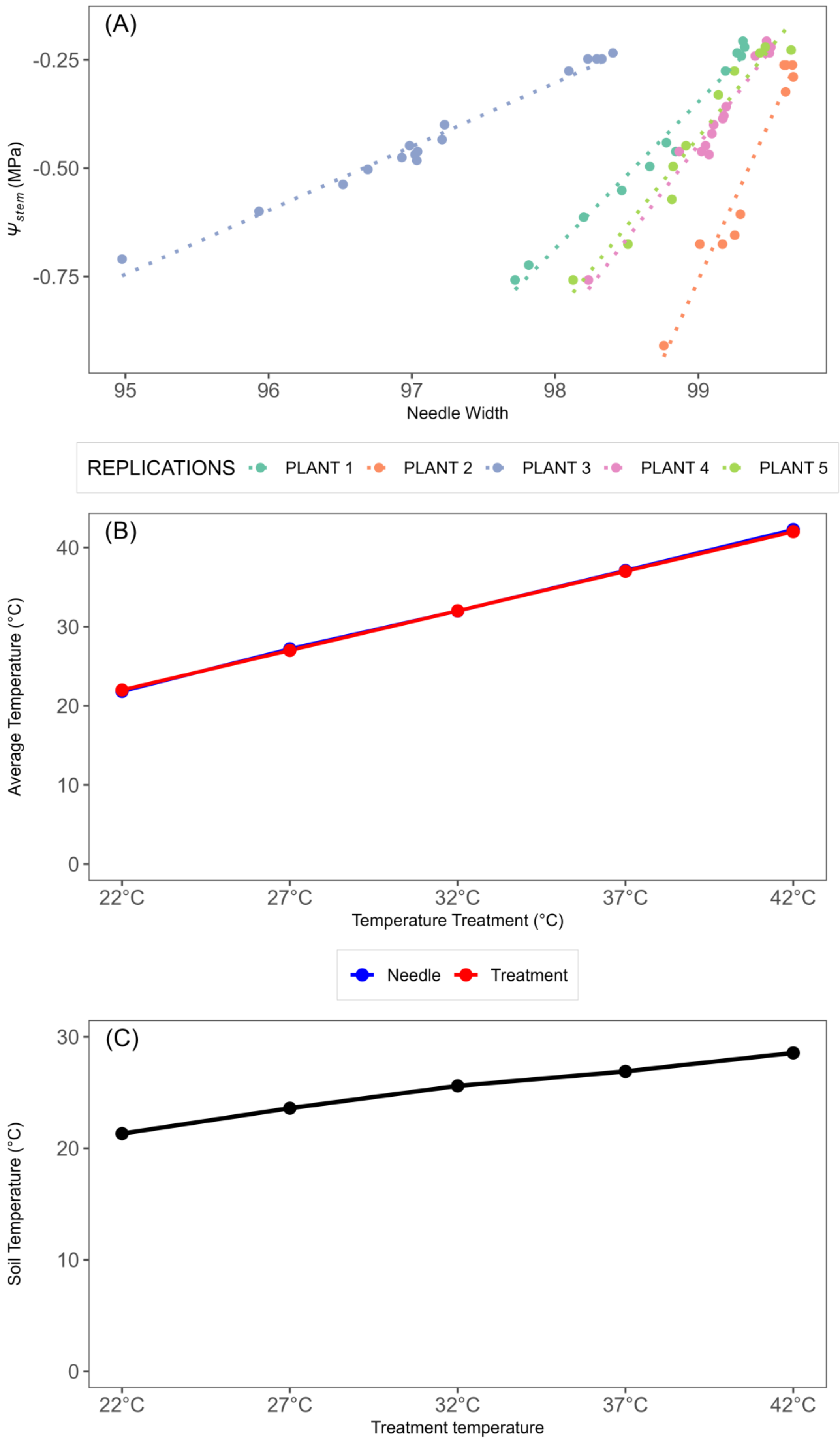
(A)
-0.25
-0.50
-0.75
Ψstem (MPa)
95
96
97
98
99
Needle Width
REPLICATIONS
PLANT 1
PLANT 2
PLANT 3
PLANT 4
PLANT 5
(B)
40
30
20
10
0
Average Temperature (°C)
22°C
27°C
32°C
37°C
42°C
Temperature Treatment (°C)
Needle
Treatment
(C)
30
20
10
0
Soil Temperature (°C)
22°C
27°C
32°C
37°C
42°C
Treatment temperature

Supplementary Fig. S3 (A) **Response of mean soil-to-stem hydraulic conductance ($K_{s\text{-}s}$, mmol m^{-2} s^{-1} MPa^{-1}) (circles in red colour) and mean $K_{s\text{-}s}$ normalized to 20ºC (circles in black colour) for each temperature treatment in all *Pinus radiata* replicates. The bars represent standard deviation across all the replicates. $K_{s\text{-}s}$ value normalized to 20ºC ranged from 11.2±3.0 mmol m^{-2} s^{-1} MPa^{-1} at 22ºC to 6.8±1.3 mmol m^{-2} s^{-1} MPa^{-1} at 42ºC and was found to be constant with no significant differences up to 37ºC ($P > 0.05$). However, a significant decline was found in $K_{s\text{-}s}$ at only 42ºC as compared to the first temperature treatment (22ºC) ($P < 0.05$). On accounting for viscosity at each temperature treatment, $K_{s\text{-}s}$ values at each temperature treatment ranged from 11.0±2.2 at 22ºC to 8.1±1.3 at 42ºC and were found to have no significant differences ($P > 0.05$).** (B) **The maximum quantum yield of Photosystem II (Fv/Fm ratio) in response to different temperature treatments. The mean Fv/Fm of detached needles (averaged over from five replicates) at 25ºC remained constant with no significant decline until 37ºC ($P > 0.05$). However, a significant decline was seen at higher temperature treatments ($P < 0.05$) with almost 0 at 52ºC. The black circles represent the mean Fv/Fm value at each temperature treatment and the bars represent standard deviation across all the replicates.**

ALT TEXT:

Supplementary Fig. S3 (A) Plot shows response of mean soil-to-stem hydraulic conductance ($K_{s\text{-}s}$, mmol m^{-2} s^{-1} MPa^{-1}) and mean $K_{s\text{-}s}$ normalized to 20ºC at each temperature treatment. $K_{s\text{-}s}$ value normalized to 20ºC showed a significant decline only at 42ºC as compared to the first temperature treatment (22ºC), however, on accounting for viscosity at each temperature treatment, $K_{s\text{-}s}$ values at each temperature treatment showed no significant differences. (B) Plot showing no significant decline in maximum quantum yield of Photosystem II (Fv/Fm ratio) until 37ºC, followed by significant decline thereafter. Replicates are illustrated with individual-coloured points.

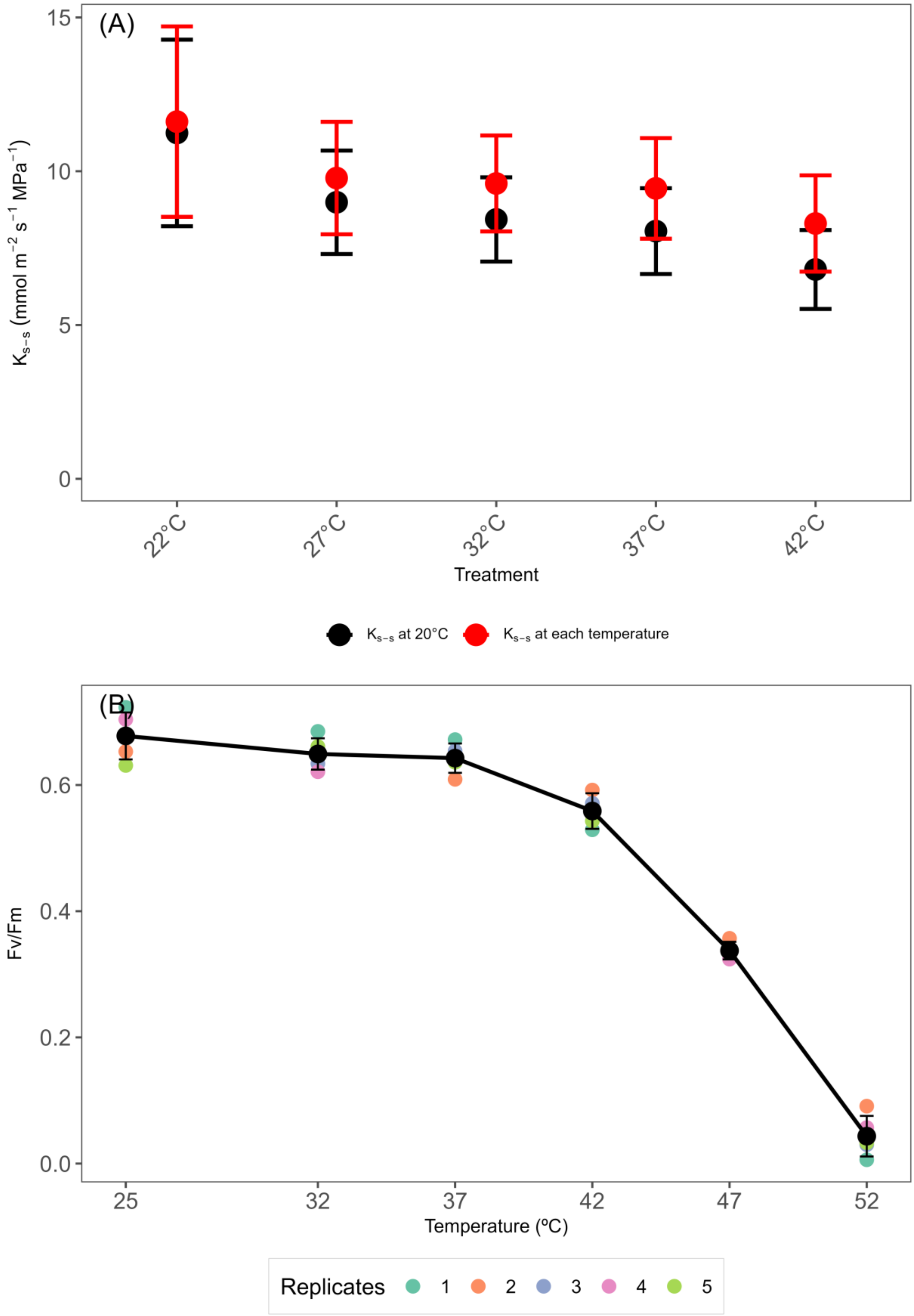
(A)
K_{s-s} (mmol m⁻² s⁻¹ MPa⁻¹)
15
10
5
0
22°C
27°C
32°C
37°C
42°C
Treatment
Ks-s at 20°C
Ks-s at each temperature
(B)
Fv/Fm
0.6
0.4
0.2
0.0
25
32
37
42
47
52
Temperature (°C)
Replicates
1
2
3
4
5
